# A Rigidity-Enhanced Antimicrobial Activity: A Case for Linear Cationic α-Helical Peptide HP(2–20) and Its Four Analogues

**DOI:** 10.1371/journal.pone.0016441

**Published:** 2011-01-24

**Authors:** Li Liu, Ying Fang, Qingsheng Huang, Jianhua Wu

**Affiliations:** 1 Institute of Biomechanics and Department of Biomedical Engineering, School of Bioscience and Bioengineering, South China University of Technology, Guangzhou, China; 2 School of life Science, Sun Yat-Sen University, Guangzhou, China; Sun Yat-Sen University, China

## Abstract

Linear cationic α-helical antimicrobial peptides are referred to as one of the most likely substitutes for common antibiotics, due to their relatively simple structures (≤40 residues) and various antimicrobial activities against a wide range of pathogens. Of those, HP(2–20) was isolated from *Helicobacter pylori* ribosomal protein. To reveal a mechanical determinant that may mediate the antimicrobial activities, we examined the mechanical properties and structural stabilities of HP(2–20) and its four analogues of same chain length by steered molecular dynamics simulation. The results indicated the following: the resistance of H-bonds to the tensile extension mediated the early extensive stage; with the loss of H-bonds, the tensile force was dispensed to prompt the conformational phase transition; and Young's moduli (N/m^2^) of the peptides were about 4∼8×10^9^. These mechanical features were sensitive to the variation of the residue compositions. Furthermore, we found that the antimicrobial activity is rigidity-enhanced, that is, a harder peptide has stronger antimicrobial activity. It suggests that the molecular spring constant may be used to seek a new structure-activity relationship for different α-helical peptide groups. This exciting result was reasonably explained by a possible mechanical mechanism that regulates both the membrane pore formation and the peptide insertion.

## Introduction

Antimicrobial peptides (AMPs), an innate immune component ubiquitous among plants and animals, are variously active against a wide range of pathogens, such as gram-positive bacteria, gram-negative bacteria, fungi and protozoa [1], [2], [3]. They are therefore proposed as one of the most likely substitutes for common antibiotics, to confront an increasingly serious threat to human health caused by antibiotic-resistant bacterial infection [4], [5], [6]. Of these, the linear cationic α-helical peptides have been extensively researched due to their relatively simple structures (≤40 residues) and accessibility to chemical synthesis [1], [7]. The linear cationic α-helical peptide HP(2–20) isolated from the N-terminal region of the *Helicobacter pylori* ribosomal protein can activate phagocyte NADPH oxidase to produce reactive oxygen species while being a neutrophil chemoattractant with bactericidal potency [8].

A profound interest has been taken in non-receptor-mediated interaction of AMPs and target cell membrane, to reveal the mechanism regulating the action and activities of AMPs [1]. It is believed that the antimicrobial activity is related to structural determinants, such as the peptide conformation, charge, hydrophobicity, amphipathicity and polar angle [9]. For the action of AMPs, a rational theme is that, as the peptides meet a target cell, the positive charges are beneficial for them to be captured and bound to the cellular membrane by electrostatic affinity [10]; the bound peptides interact with the cellular membrane by their hydrophobic face [11], and may undergo a conformational phase transition in the framework of the cellular membrane via electrostatic, hydrophobic or other affinities [9]; but, the membrane pore or channel formation, which causes dysfunction of the cell, occurs just as the accumulation of the bound peptides on the cellular membrane has arrived at a stoichiometric threshold [12]; and then, the membrane disruption is induced, or the peptides would directly enter the membrane to access and inhibit intracellular targets [1], [9]. However, previous works were focused mainly on biochemical and biophysical aspects instead of mechanical correspondence in the interaction of the peptides and cellular membrane.

In contrast, intuitively there may be a mechanical mechanism to regulate the action of AMPs. It was indicated that, the flexibility induced by the hinge sequence in the central part of the peptides would allow the α-helix in the C-terminus to closely span the lipid bilayer, and increase the antimicrobial activities, while the deletion of the hinge sequences will decrease the bactericidal rate significantly [13], [14], [15]. The enhanced rigidity of the red cell membrane bound with ligands [16] hints that, the rigidity of cellular membrane also may increase remarkably with the accumulation of the bound peptides, and then regulate the stretching and bending as well as the disruption of the membrane under loads. On the other hand, a stable structural conformation, which may be required for the interaction of AMP and membrane [17], [18], refers to the spring constant of the peptide, and the conformational phase transition nearly always occurs in a mechanical environment. Besides, rigidity requirement is exhibited in many biological processes. For instance, in maintaining cell shape or aiding cell movement, a modest range of spring constant is required for cytoskeleton and diverse filaments in a cell [19]; the protein structure with an adequate rigidity may provide a foothold for the activation process of muscle contraction [20]; and, a rigid conformation for an enzyme molecule is required to hold its substrate in an activated conformation [21]. From these, it comes that the complex process involved in the action of AMPs may be rigidity-dependent, similar to the important roles of the mechanical properties of biomolecules in numerous biological processes.

Many efforts in biomechanical measurements at single-molecule level had been taken in the recent years [22]. In these experiments, the molecular tensile strengths were examined from the force-extension curves of the molecules that were stretched by means of ultrasensitive force instruments, such as atomic force microscopy [23] and optical tweezers [24]. Numerical prediction of the elasticity of protein or polypeptide via steered molecular dynamic (SMD) simulation was shown to be highly consistent with the data of the experimental measurement [25]. More and more knowledge have been obtained on the mechanical properties of biomolecules, such as DNA [26], RNA [27] and protein molecules [28], [29], [30], but less on AMPs. The lack of the knowledge on the mechanical properties of peptides greatly limits what can be learned about the possible mechanical mechanism that regulates the action of AMPs. There is moreover an experimental barrier in examining the rigidities of extremely short peptides, such as the linear cationic α-helical AMPs of ∼3 nm. Besides, little published structural data can be used to estimate the extensive elasticities of the peptides by SMD simulation.

However, it should be suitable to pay our attention to the linear cationic α-helical AMPs for their simple α-helical structures firstly, in order to verify whether or not there is a mechanical mechanism regulating the action of the peptides. Here we chose HP(2–20) and its four analogues, HPA1, HPA2, HPA3 and HPA5, as a simple illustrative case. It was indicated that the action of HP(2–20) has different features not only in the induced pore sizes of the cellular membrane but also in the antimicrobial activities against *C*. *albicans*, *S*. *aureus*, *E*.*coli* and so on, comparing with its different analogues [8]. Here, we investigated the unfolding of the peptides by SMD simulations, examined the tensile responses of the peptides in resisting their conformational phase transitions especially in the early stage of extension, and estimated their spring constants. By relating the estimated spring constants of these peptides with the published data [8], [31] of the minimum inhibition concentrations (MICs) aimed at *C*. *albicans*, *S*. *aureus* and *E*.*coli*, respectively, a possible rigidity-enhanced activity for these peptides was exhibited. Our exciting results provided a possible mechanical interpretation of the action of these peptides, and a clue to develop a new activity design method by making the analogue harder or softer than its template.

## Results

### Tensile Responses and Conformational Variations of the α- helical Peptides

**Figure 1:**
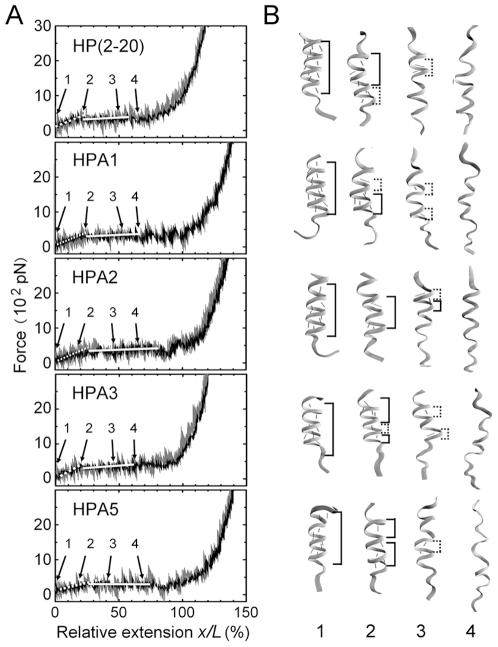
Tensile force *F*–relative extension *x/L* curves (*A*) with four representative conformation snapshots (*B*) for HP(2–20), HPA1, HPA2, HPA3 and HPA5 respectively. The weight lines (*A*) express the mean *F*–*x/L* curves, in which both the tensile force and the relative extension are means over five independent stretching events with corresponding different initial conformations for each of these peptides. The light lines (*A*) are the instantaneous *F*–*x/L* curves to express respective tensile events for each peptides, and the number marks (*A* and *B*) from 1 to 4 on the curves are used to denote different stretched states of the peptides, being initial, elongated with intact helical chain, unfolded partly and totally, respectively. The accompanied conformation snapshots (*B*) in the right hand of the *F*–*x/L* curves are selected from these different stretched states, respectively. The two different structural parts along the chain, the α-helical and 3_10_-like helical zones, so called for their respective H-bonds from *i*-th to (*i*+4)-th residue and from *i*-th to (*i*+3)-th residue, are marked (*B*), respectively. The main-chain H-bonds are represented by red dashed lines (*B*). The values of the local spring constants can be read from the slopes (*A*, white lines) of the *F*–*x/L* curves in different extension regions.

We simulated the unfolding processes of the peptide HP(2–20) and its four analogues, HPA1, HPA2, HPA3 and HPA5, by SMD with pulling velocity of 0.01 nm/ps and time step 2fs, to probe the tensile responses of these peptides (see Materials and Methods). The instantaneous and mean *F*–*x/L* curves were respectively exhibited by the gray and dark lines in Fig. 1 *A*, which were accompanied with the conformational snapshots (Fig. 1 *B*) selected from different stretched states of these peptides. The results illustrated that, the nonlinear and irregular characters of the instantaneous *F*–*x/L* curves were so strong that it was difficult to examine quantitatively the mechanical properties of the peptides. These ambiguous mechanical responses might come from different initial conformations and three irregular complex processes, such as local denaturation of the α-helical chain and response to the thermal excitation as well as the irregular breaking and reemerging of H-bonds; but the mechanical properties of these peptides emerged more obviously from their corresponding mean *F*–*x/L* curves (the dark lines in Fig. 1 *A*), in which both the tensile force *F* and the relative extension *x/L* were means over five different stretching events for each peptides. From the *F*–*x/L* curves, it comes that, the tensile force increases as relative extension increases in the early stage of stretching, and follows a “pseudoplateau” over a wider range of relative extension after passing the inflection point of *x/L* with values of about 0.2, then increases rapidly as the peptides are further stretched. These nonlinear properties of the *F*–*x/L* curves are similar to the results examined by AFM for the alanine-based α-helical polypeptide [32], and consistent with the theoretical argument proposed by Chakrabarti and Levine [33].

The above tensile features of the α-helical peptides would be related closely to a phase transition from an α-helical to an extended conformation. The conformational snapshots (Fig. 1 *B*) in different unfolding stages indicated that, upon stretching, the peptides firstly kept their α-helical conformations, then suffered a conformational phase transition from α-helical H-bonds to 3_10_-like helical H-bonds; and accompanied with the decrease of H-bonds, this conformational phase transition would be carried out until the peptides had been unfolded totally. This observation is in good agreement with those predicted theoretically by Rohs et al [34] and then observed by Afrin *et al* [32]. Besides, from both *F*–*x/L* curves (Fig. 1 *A*) and conformational snapshots (Fig. 1 *B*), we observed that, for each of these peptides, the unfolding starts at C- or N-terminal, but the tensile force that mediates the passive denaturation of local helices along the peptide seemed obviously different. It implies the mechanical responses to the tension are sensitive for these peptides with just one or two different residues.

### Survival ratios of H-bonds linearly decline with extension of the Peptides

**Figure 2:**
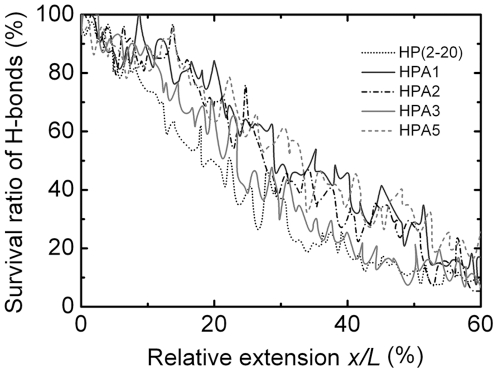
Variation of survival rate of main-chain H-bonds versus the relative extension *x*/*L* for HP(2–20), HPA1, HPA2, HPA3 and HPA5, respectively. The survival rate of main-chain H-bonds is a mean of the survival ratios of main-chain H-bonds recorded in five unfolding events of different initial conformations in equilibrium for each of the peptides.

In the processes of such conformational phase transitions shown in Fig. 1 *B*, two key events, the breaking of H-bonds and the occurring of the local denaturation of main chain were interrelated for respective peptides. In SMD simulations, we recorded and then averaged the survival ratios of the main-chain H-bonds in five tensile unfolding events of different initial conformations in equilibrium for each peptide. The variation of the survival rate of the main-chain H-bonds versus the relative extension were shown in Fig. 2. The results indicated that, the increasing of the relative extension monotonously linearly decreased the survival ratio of the main-chain H-bonds with different decline rates, which were read to be approximately 2.1, 1.5, 1.8, 2.1 and 1.6 in the region of 0<*x/L*<0.4 for peptide HP(2–20), HPA1, HPA2, HPA3 and HPA5, respectively; these survival ratios at a fixed *x/L* lay in different levels for different peptides, respectively, and the decreasing of the survival ratio of the main-chain H-bonds still allowed about 50∼75% of H-bonds being maintained as the relative extension arrived at the value of 0.2.

The implication of the various survival ratios of H-bonds is that there may be different mechanical response properties for these peptides. As H-bonds broke, the resistance to stretching would be provided gradually by the main chain, whose local denaturation might occur firstly at C-terminal or N-terminal and then spread to other region (Fig. 1 *B*); the higher the level of the survival ratio of main-chain H-bonds, the more the resistance of the main chain to the extension. The resistance of the H-bonds to the tensile extension would mediate the early-middle stage of stretching process for each of the peptides, and, with the losing of the H-bonds, the tensile force would be mainly dispensed to prompt the conformational phase transition of the peptides gradually[32]. The above results suggest that the main-chain H-bonds will prompt the conformation stabilization, perhaps explaining why the slopes of the *F*–*x/L* curves (Fig. 1 *A*) in the early stretching stage are larger than those in the “pseudoplateau” range, because a larger slope means a larger resistance to the tensile extension.

### Molecular rigidities and their sensitive dependence on the residue compositions

**Figure 3:**
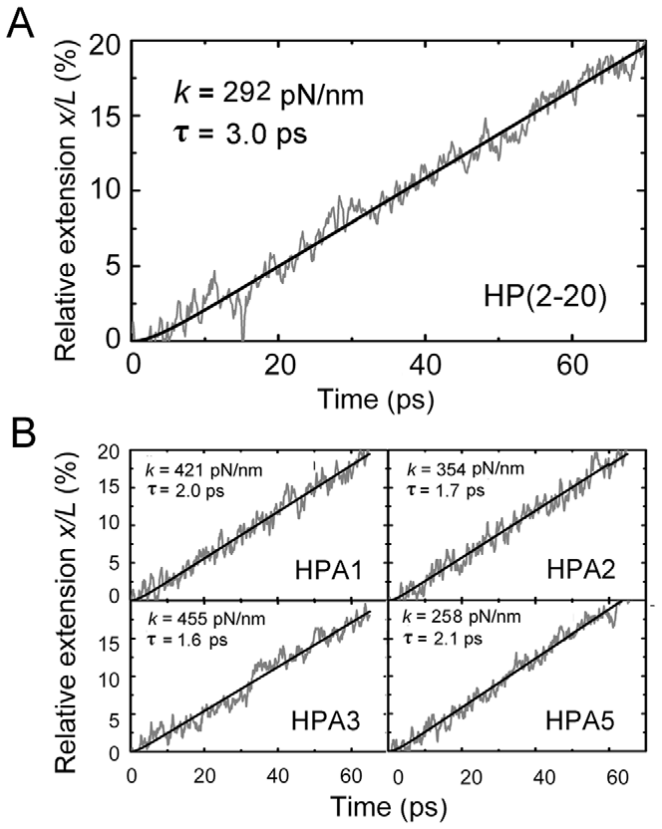
Comparison of instantaneous (irregular curves) and fitted (smooth lines) time courses of the relative extension *x/L* in the region of *x/L* from zero to 0.5 for HP(2–20) (*A*) and its four analogues (*B*). The representative instantaneous time courses of *x/L* were recorded from their corresponding tensile events simulated by SMD with pulling velocity of 0.01 nm/ps and time step 2fs. The predicted time courses of *x/L* came from Eq.2, the solution of Langevin equation, with respective best fitting values (*A* and *B*) of spring constant *k* and relaxation *τ*. The simulated and fitted *x/L*–*t* curves are in well agreement with each other, except for small *t* values less than about 2*τ*.

Here we mainly focused on the mechanical properties of the peptides in region of *x/L* from zero to 0.2. The reason is that, a bound peptide should have only a small extension to correspond with the deformation of the target cell membrane, so that, for the mediation of antimicrobial action, the mechanical property of the peptide in the relative extension region of 0<*x/L*<0.2 should be more important than that in other regions,. For comparison, two methods based on Hook's law and Langevin equation (see Material and Methods) were used to extract the values of the spring constant *k* in the region of 0≤*x/L*≤0.2 from five independent stretching events, which were simulated by SMD with respective initial conformations in statistic equilibrium for each peptides. The validity of the method base on Hook's law emerged from the linear variation of the tensile force *F* versus the relative extension *x/L* less than 0.2 (Fig. 1 *A*), and, as shown in Fig. 3 *A* and *B*, the time courses of *x/L* simulated by SMD were in good agreement with that predicted by the theoretical solution (Eq.2) of Langevin equation with corresponding best fitting values of the relaxation time *τ* and the spring constant *k*.

**Figure 4:**
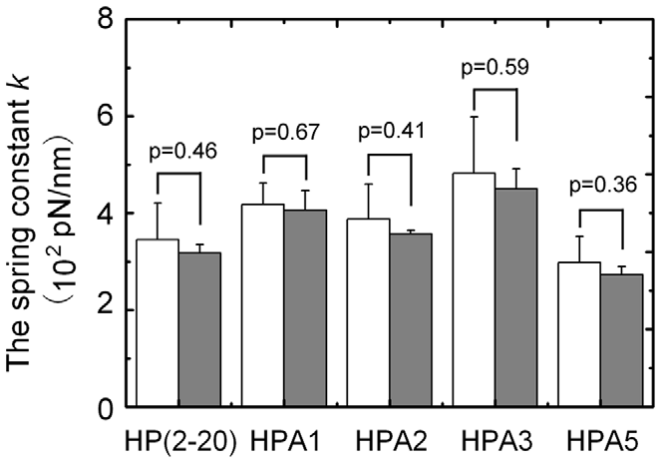
Comparison between values of spring constant *k* (white bars) read from slopes of *F*-*x/L* curves (**Fig. 1 ***A*****) and those (gray bars) estimated by fitting *x/L*-*t* curves (**Fig. 3**) with Langevin equation (Eq. 1) in the region of *x/L* from zero to 0.2 for HP(2–20), HPA1, HPA2, HPA3 and HPA5, respectively. The data are presented as mean ± SE of five different values of *k* extracted from five independent stretching events for each peptide. There were no statistically significant differences in the values of *k* derived from the two different methods, due to *p*-values ranging from 0.36 to 0.67 in student *t*-test, for each of the peptides. The statistical differences in *k* values among these peptides were similar to those in their corresponding Young's moduli (Fig. 5).

Fig. 4 presented the values of *k* expressed as mean±SE of the data, which were estimated from simulated curves of *F*–*x/L* (Fig. 1 *A*) and *x/L*–*t* (Fig. 3 *A* and *B*), respectively, in the region of 0≤*x/L*≤0.2. The results illustrate that, for each of the peptides, *k* value has a deviation of about 20% from the mean, that is, the peptide will exhibit different mechanical behaviors, when it is being stretched from its different initial conformations in equilibrium; there is no remarkable statistical differences in the *k* values from the two methods, but the *k* value may be over-estimated about 10% in disregard of damping effect of the water molecules; in contrast, significant statistical differences exist for the *k* values of different peptides, and would gradually increase by 50% along the way of HPA5 → HP(2–20) → HPA2 → HPA1 → HPA3, that is, the spring constants of the peptides would be closely related to their residue compositions. Moreover, it suggests that, for a linear cationic α-helical antimicrobial peptide, its mechanical properties is sensitively dependent on its residue composition, and with only one or two residues being replaced, the spring constant of the peptide may increases or decreases conspicuously. By comparing the spring constant data of 0.2–15 pN/nm for PGA [35], poly-L-Lys [36], apocalmodulin [37] and C(KAAAA)_10_KC]_n_ [32] at 50% of chain extension, respectively, our *k* values of 200–500 pN/nm (Fig. 4) are higher for 1–2 orders. This inconsistency lies mainly on the different regions of chain extensions selected to fit the *F*–*x/L* curves, and disappears as extracting the *k* values in a moderate relative extensive region involved in the “pseudoplateau” of the *F*–*x/L* curves. In fact, we had read the values of *k* in the region of 0.2<*x/L*<0.6 to be about 4.8, 9.5, 12.9, 17.6 and 8.0 pN/nm for HP(2–20), HPA1, HPA2, HPA3 and HPA5.

**Figure 5:**
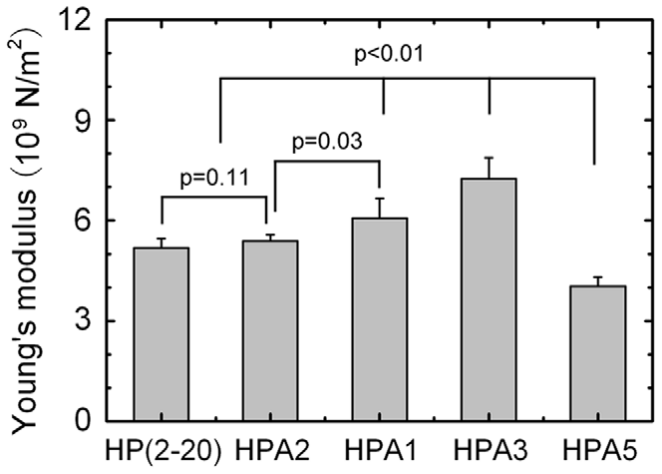
Comparison of Young's modulus *E* of HP(2–20), HPA1, HPA2, HPA3 and HPA5. The data of *E* are presented as mean ± SE of five different values derived from the *k* values, the best fitting results of *x/L*-*t* curves of five independent stretching events for each peptide. Significant differences (*p*-values ranging from 0.01 to 0.03) lie in the *E* values among these peptides, except for the difference (*p* = 0.11) in the *E* values of HP(2–20) and HPA2.

Using the spring constant data and modeling each of the peptides as a circular rod with radius of 0.25 nm and length listed in Table 1, we estimated Young's moduli of the peptides by *E* = *kl/A*, where *l*, *A* and *E* are the length, cross-sectional area and Young's modulus of the peptide, respectively. The results were plotted in Fig. 5, which indicated Young's moduli of the peptides to be 4∼8×10^9^ N/m^2^. Our results showed there was a significant difference (*p*-values ranging from 0.01 to 0.03) in the *E* values among these peptides, except for the difference (*p* = 0.11) in the *E* values of HP(2–20) and HPA2. The statistical differences in values of *k* were similar to those in their corresponding Young's moduli, as it should be.

**Table 1:** Sequences, Contour Lengths, and MICs of HP(2–20) and Its Four Analogures.

AMP	Sequence*	MIC (µM)						Contour length‡ (nm)
		*C.albicans*		*S.aureus*		*E.coli*		
		Ref.31	Ref.8†	Ref.31	Ref.8†	Ref.31	Ref.8†	
HP(2–20)	AKKVFKRLEKLFSKIQNDK	>25	25	12.5	6.25	3.13	6.25	3.20±0.05
HPA1	AKKVFKRLEKLFSKIQN*W*K	25	25	3.13	3.12	0.78	3.12	2.93±0.03
HPA2	AKKVFKRLEKLFSKI*W*NDK	25	25	6.25	3.12	3.13	3.12	2.96±0.07
HPA3	AKKVFKRLEKLFSKI*W*N*W*K	12.5	6.25	1.56	0.78	1.56	1.56	3.16±0.06
HPA5	AKKV*S*KRLEKLFSKIQNDK	>25	>100	>12.5	50	6.25	6.25	2.91±0.05

*The italic letters in the sequence column indicate the substituted amino acid residues;

† The MIC of bacterial growth were measured in low-salt buffer;

‡ The original contour lengths were presented as mean±SE of five different lengths measured from five random conformations in equilibrium for each peptide.

### A harder peptide has a stronger antimicrobial activity

**Figure 6:**
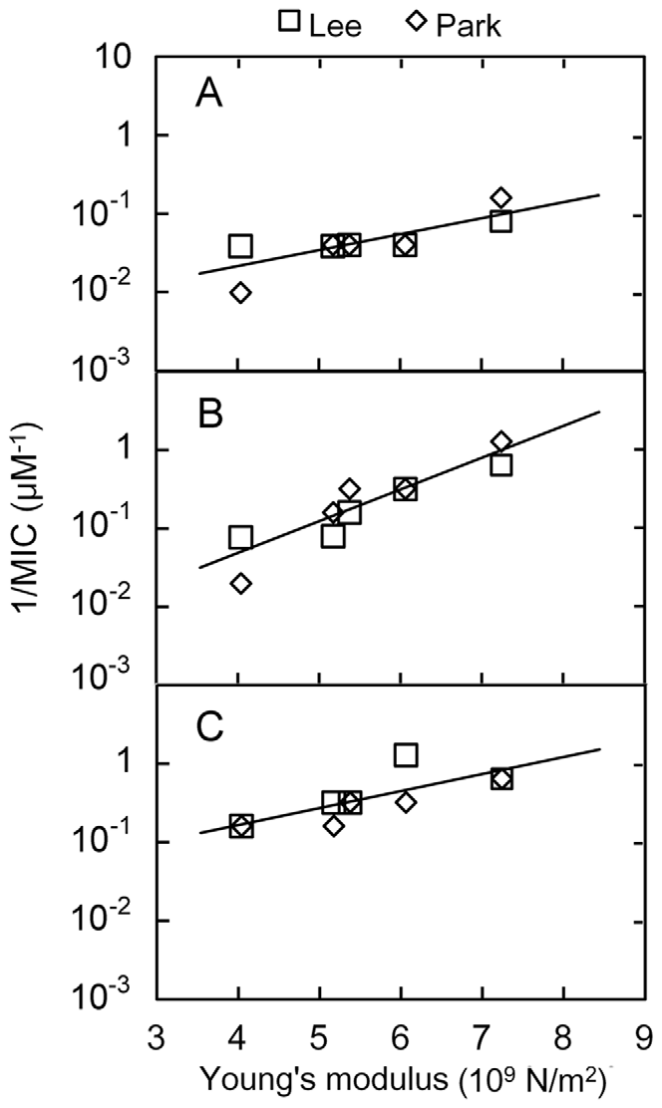
Variation of reciprocal of minimum inhibition concentration (MIC) against Young's modulus *E* for HP(2–20), HPA1, HPA2, HPA3 and HPA5. Here, the value of *E* is the mean of five best fitting results derived from five independent stretching events for each peptide (Fig. 5). The reciprocal of MIC is used to evaluate the antimicrobial activity for each of the peptides. The data of MICs aimed at *C.albicans, S.aureus* and *E.coli* are taken from previous works (see Table 1), which were respectively reported by Lee[31] and Park[8] *et al*.

Mechanically, the rigidity not only represents the deformational ability of a peptide but also may govern the conformational phase transition of the peptide under loads. Thus, besides the structural parameters, such as conformation, hydrophobicity, amphipathicity, charge, polar angle and so on [9], rigidity may be a determinant that regulates the activities of the linear cationic α-helical antimicrobial peptides. To examine this hypothesis, we plotted the Young's moduli of the peptide HP(2–20) and its analogues against the reciprocals of the data (Table 1) of their corresponding MICs aimed at *C*. *albicans*, *S*. *aureus* and *E*.*coli*, as shown in Fig. 6, where the values of the Young's moduli came from the *k* values estimated by fitting the time courses (Fig. 3) of the relative extension *x/L* in the region of 0<*x/L*<0.2, and the two sets of the MIC values were taken from the previous published data [8], [31].

As predicted, the results (Fig. 6) demonstrate that, for each of the peptides aimed at *C*. *albicans*, *S*. *aureus* and *E*.*coli*, the reciprocal of the MIC is proportional to the Young's modulus, i.e. the MIC decreases almost monotonously as the Young's modulus increases. It means that, the larger the Young's modulus, the stronger the antimicrobial activity, that is, a harder peptide has a stronger antimicrobial activity. Even though there are differences between the two sets of the MIC data, this rigidity-dependent antimicrobial behavior is present among all of *C*. *albicans*, *S*. *aureus* and *E*.*coli*. The different slopes of the fitting lines in Fig. 6 illustrate that, against *C*. *albicans*, *S*. *aureus* and *E*.*coli* in turn, this rigidity-enhanced antimicrobial activity would become more and more remarkable.

This novel possible rigidity-enhanced antimicrobial activities of HP(2–20) and its four analogues elaborated above implies that, for a linear cationic α-helical antimicrobial peptide, its spring constant would be regarded as a macroscopic measurement index, which may have synthesized the effects of the structural parameters on its antimicrobial activity. A possible explanation for the phenomenon comes from a rigidity-dependent mechanism that regulates the action of the peptide (see Discussion).

## Discussion

As mentioned above, the antimicrobial activities of HP(2–20) and its four analogues may be rigidity-enhanced. It implies there is a rigidity-dependent mechanism that regulates the action of the peptides. A rational explanation for this possible mechanical mechanism would be referred to both the pore formation and the peptide insertion, the two pivotal events in the nonspecific interaction of the α-helical peptides and the target cell membrane [1], [9].

Intuitively, there should be a mechanical mechanism that regulates the formation of the pore or slot in cellular membrane. It had been indicated that, when free peptides meet a bacteria, they will first be captured and bound to the target cell [10], and then the cellular membrane would be divided into two different parts, namely the peptide-free and the peptide-bound leaflets [38], in which the lipids are either free of or associated with the peptides. Mechanically, the elastic modulus of the peptide-bound leaflets would be larger than that of the peptide-free leaflets, because the bound peptides on the membrane were like crusted patches, which would not only limit the undulation of the bound lipids but also weaken the elastic deformability of the peptide-bound leaflets. It illustrates that there would have a step variance of the elastic modulus over the edge of a crusted patch for the bound membrane. Through accumulation of the bound peptides, the elastic modulus of the membrane would not only increase, just as the case of the red cell bound with ligands [16], but also become more and more non-uniform. Thus, under active or passive movement of the cellular membrane, the transient or prolonged stress concentration would occur at the edges between peptide-free and peptide-bound leaflets, and then facilitate the formation of some slots or pores in the membrane. Obviously, a more rigid bound peptide would imply a stronger constraint to the longitudinal and transversal fluctuations of the bound lipids in a peptide-bound leaflet, leading a more significant stress concentration to occur at the edge of the peptide-bound leaflet, and further making the peptide-bound leaflet be torn more easily away from the cellular membrane. It may provide a mechanical explanation for the observation that, prior to pore formation, the bound peptides would decrease the order of the adjacent lipids and increase the fluctuation in the bilayer thickness [38], [39].

An elastic modulus gradient pulse may exist in the target cell membrane bound with the α-helical peptide HP(2–20) and its four analogues. By regarding Young's modulus of the membrane/peptide complex as the sum of the moduli of both the lipid bilayer and the peptides, the elastic modulus non-uniformity of the cellular membrane binding with the peptides can be evaluated approximately. For instance, the average Young's modulus for hydrated and dried *E.coli* were reported to be 0.25∼0.45 and 3.7∼4×10^8^ N/m^2^, respectively, in the sacculus axis orientations perpendicular or parallel to grooves of the cell [40]. By comparison with the Young's moduli (Fig. 5) for the peptide HP(2–20) and its four analogues, we obtained the modulus of the membrane of the dried or the hydrated *E.coli* would be one or two orders of magnitude lower than that of the peptides, alluding that, over the edge of a peptide-bound leaflet, there would have ten folds variance of Young's modulus of the membrane for *E.coli*.

Besides, there may be another rigidity-dependent mechanism in entering the target cell membrane for these α-helical peptides. Generally, a stable structure is required for some peptides reaching and entering into the membrane [17], [18], that is, the peptides should not be too soft for the realization of antimicrobial activity. Comprehensibly, as inserting into the pore or slot in the membrane, a rigid slender rod should meet fewer barriers than a limp one.

The above discussion on both the membrane pore or slot formation and the peptide insertion seems to be a rational argument for the rigidity-enhanced antimicrobial activities of HP(2–20) and its four analogues (see Fig. 6). In contrast, the antimicrobial activity and the selective toxicity are related to the inducibility of an α-helical conformation in a membrane-mimicking environment, rather than intrinsic helical stability for some peptides. For instance, in short cecropin/melittin hybrid analogues with lysine and glutamine residues placed so as to form lactam bonds in a helical conformation, the antimicrobial activity of such preformed helical peptides was considerably reduced [41]; in addition, different conformations may be formed as the compositions of target membranes are different [9], [42]. Furthermore, a soft hinge structure would increase antimicrobial activity markedly [13], [14], [15]. These mean that the rigidity-enhanced activity may only exhibit a face of a coin of elasticity-dependent antimicrobial mechanism for the linear cationic α-helical antimicrobial peptides. Mechanically, in the antimicrobial process, the captured peptides should be allowed to the conformational alteration in the framework of the target cell membrane, and then contacted closely to the membrane. A harder peptide should have a more stable structure, but a softer one has a better deformability to fit the membrane framework. Both the structural stability and the deformability are related to the antimicrobial activity of the peptide, and a fine balance is needed for the peptide to interact with and exploit vulnerabilities inherent in the target cellular membrane.

Doubtlessly, well-grounded rigidity values are required for revealing whether or not there is a rigidity-dependent antimicrobial mechanism for a linear α-helical peptide group. Despite that biomechanical measurement of single molecule can be performed [23], it is still a challenge to measure experimentally the spring constant of a short peptide of ∼3 nm. For these reasons, here we perform SMD simulation to evaluate the mechanical properties of HP(2–20) and its four analogues by two methods based on Hook's Law and Langevin equation [25], respectively. To filter out the random noises in the mechanical responses, five unfolding events were observed by SMD simulation with different initial conformations for each peptide. Our results illustrated that, the two methods used here are substitutable, because their corresponding values of spring constant *k* has no significant statistics difference (Fig. 4). The reason may be that, the damping effect of water molecules on the mechanical response of the tensile extension lies just in the early stretching stage of 0<*t*<3*τ.* In fact, from Eq. 2, we found that, the best fitting values of the relaxation time *τ*, which were extracted from simulated time courses of extension (Fig. 3 *A* and *B*), were about 1.5∼3.2 ps, corresponding to the relative extension *x/L* of about 1% for HP(2–20) and its four analogues. As mentioned above, our *k* values of 5–18 pN/nm at 20∼60% of chain extension are comparable with the spring constant data of various polypeptide molecules in previous works [35]. It suggests that, SMD simulation is an effective tool in quantitatively examining the mechanical properties of such short α-helical peptides.

Here, we focused our attention on the mechanical properties of such α-helical peptides in the early stage of unfolding, because just a small extension would be involved in the interaction of the cellular membrane and a bound α-helical peptide. Our results demonstrated that, there are statistics differences (Fig. 5) in the rigidities of HP(2–20) and its four analogues. This sensitivity of the mechanical properties to the residue composition should be related closely to a complex process of both the H-bond breaking and the local main-chain denaturation, which regulate the conformational phase transition. Indeed, there are many unknowns in this process, but the resistance of H-bonds to the extension mainly lies in the early stretched stage for such α-helical peptides.

However, the possible rigidity-enhanced antimicrobial activities of HP(2–20) and its four analogues suggest that, for a linear cationic α-helical antimicrobial peptide, its spring constant can be regarded as a macroscopic measurement index, which may have synthesized the effects of other structural parameters on its antimicrobial activity and be used to seek for a new structure-activity relationship. Besides, our results also provide a possibility to develop a new mechanical design method of the antimicrobial activity by making the analogue to be harder or softer than its template of α-helical peptide.

## Materials and Methods

### Antimicrobial Peptides and Their Minimum Inhibition Concentration

We here chose the AMP HP(2–20) and just its four corresponding analogues, namely HPA1, HPA2, HPA3 and HPA5, from their template with one or more residues being replaced [8], [31]. These peptides all are linear cationic α-helical peptides with same chain length of nineteen residues, and can inhibit the growth of *C. albicans*, *S. aureus*, *E. coli*, etc. The minimum inhibition concentrations (MICs) aimed at *C. albicans*, *S*. *aureus* and *E*.*coli* were used to assess antimicrobial activities of these peptides. Naturally, a smaller MIC value means a stronger activity. Thus, the reciprocals of MICs would reflect the antimicrobial activities of the peptides directly. We listed the values of both the sequences and MICs against *C. albicans*, *S*. *aureus* and *E*.*coli* in Table 1. These data were taken from the previous published works [8], [31], in which the antimicrobial assays of these peptides were described in detail.

### Steered Molecular Dynamics Simulation

We simulated numerically the unfolding of the peptides by the steered molecular dynamics (SMD) modeling techniques. The crystal structures of the peptide HP(2–20) and its analogues, HPA1, HPA2, HPA3 and HPA5, were obtained from their PDB files from the RCSB Protein Data Bank entry 1P0G, 1P0J, 1P0L, 1P0O and 1P5L, respectively. Two software packages, visual molecular dynamics (VMD) for visualization and modeling [43], and NAMD program for SMD simulations [44], were used in the simulations. Each of the peptides was solvated with TIP3P water molecules in a rectangular box of 9 nm×4 nm×4 nm, approximately, i.e., it was surrounded by a water layer of 1 nm in each direction, except 5 nm thickness of water in direction of extension *x* for requirement of SMD simulations. The systems were neutralized by adding appropriate number of Na^+^ and Cl^−^ ions. Periodical boundary condition, along with particle mesh Ewald algorithm for electrostatic interaction and a 1.2 nm cutoff for electrostatic and van der Waals interaction, was used to perform SMD simulations with the CHARMM22 all-atom force field for protein [45]. Each system was energy-minimized for 2,000 steps, and then was equilibrated for a time period ranging from 200 ps to 500 ps, which were dependent on the responding of the peptide. SMD simulations were run on the equilibrated systems with the C-terminal C_α_ atom being fixed and N-terminal C_α_ atom being steered. The pulling was performed with time step 2fs and a constant velocity of 0.01 nm/ps, along the line between C-terminal C_α_ atom and N-terminal C_α_ atom. The virtual spring, connecting the dummy atom and the steered atom, had a spring constant *k*_1_ equal to 4863.5 pN/nm. Five stretching events were simulated with five different initial structures taken from each peptide in equilibrium.

The helicity of each peptide in equilibrium was scored by the main-chain H-bond formation criteria, which includes a distance between the O atom on the *i*-th residue and the N atom on the (*i*+4)-th residue of less than 0.34 nm, and an O-H-N angle of between 0–50°[32]. This *i*-th to (*i*+4)-th H-bond is also called as α-helical H-bond, whereas the *i*-th to (*i*+3)-th H-bond is named by 3_10_-like helical H-bond[32]. The contour length of the peptide was defined by the distance between C-terminal C_α_ atom and N-terminal C_α_ atom for each one of the peptides. The original contour lengths of the peptides were measured from their five random conformations in equilibrium, and listed in Table 1.

### Evaluation of the Spring Constants of the Peptides

In SMD simulation, the tensile force *F* and extension *x* of each of the peptides were detected as the peptide was being stretched gradually from its initial state. Then the variation of *F* versus *x* was recorded, and *k*, the spring constant of the peptide, was read from the slope of *F*–*x* curve with the use of Hook's Law *F* = *k*×*x*. Modeling the peptide as a circular rod with original contour length of *L* of ∼3 nm and radius of *a* of 0.25 nm, we estimated *E*, the Young's modulus of the peptide, by *E* = *kL*/*A*, where *A* is the cross-sectional area of the rod.

To account for the damping effect of water molecules on the mechanical response of a stretched peptide especially in beginning of the jump-ramp SMD simulation, we also used Langevin equation to model the stretching process [25]. That is, regardless of the inertial force and the thermal excitation, the extension *x*(*t*) of the stretched peptide would be governed by Langevin equation as follows:
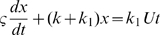
(1)with imposed initial condition *x*(0)  = 0. Here, *t* is the time, *ζ* the friction coefficient, and *U* the pulling velocity. The time course of *x*, or the solution of Eq.1, can be expressed by 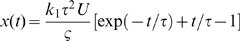
(2)


Here, *τ* is the relaxation time of the system associated with conformational changes of the peptide, *τ* = *ζ*/(*k*+*k*_1_). The above solution (Eq. 2) shows that, as time passes through a value of 3*τ*, the theoretical *x/L*–*t* course may become a straight line with just an error of 2.5%, that is, the damping effect on the extension lies in the time region from zero to 3*τ*. The validity of Eq.1 to describe the time-course of extension was shown in Fig. 3, *A* and *B*, which indicated that, with the corresponding best fitting values of the relaxation time *τ* and the spring constant *k*, the theoretical *x/L*–*t* course is in good agreement with that by SMD simulation for all of HP(2–20) and its four corresponding analogues. So, here we extracted the values of *k* and *τ* by using above theoretical time-course of *x*(*t*) to fit the *x*–*t* curves from SMD simulations of the respective tensile events.

To filter out the effect of random noises, such as the Brown motion of water molecules and the irregular breaking of amide H-bonds, on stretching of the peptides, we had observed five tensile events via SMD simulation with respective different initial conformations in equilibrium for each of the peptides. The values of *k* were expressed as the mean and standard deviation of five different spring constant data extracted from the corresponding five tensile events for each peptide.
